# Long-term efficacy and progression patterns of paclitaxel plus cisplatin and 5-fluorouracil induction chemotherapy for locally advanced, borderline-resectable esophageal squamous cell carcinoma: results from a phase II NEOCRTEC1601 study

**DOI:** 10.1097/JS9.0000000000002360

**Published:** 2025-03-28

**Authors:** Jia-Di Wu, Zhi-Qiang Wang, Qiao-Qiao Li, Chao Ren, Sheng Huang, Cai-Yan Fang, De-Shen Wang, Ji-Yang Chen, Qiong Tan, Yu-Hong Li, Hong Yang

**Affiliations:** State Key Laboratory of Oncology in South China, Guangdong Provincial Clinical Research Center for Cancer, Sun Yat-sen University Cancer Center, Guangzhou, PR China

**Keywords:** borderline-resectable esophageal squamous cell carcinoma, R0 resection, TPF

## Abstract

**Introduction:**

The NEOCRTEC1601 trial aimed to evaluate the efficacy and safety of paclitaxel in combination with cisplatin and 5-fluorouracil (TPF) as an induction chemotherapy for borderline-resectable esophageal squamous cell carcinoma (BR-ESCC). This study presents an updated 5-year analysis to further elucidate the impact of TPF chemotherapy followed by surgery.

**Method:**

This study was conducted as a single-center, phase II clinical trial. Eligibility was extended to patients diagnosed with BR-ESCC, characterized by a primary tumor or bulky lymph nodes with potential invasion into adjacent organs. The treatment protocol commenced with TPF chemotherapy, followed by surgery, if the tumor was deemed resectable, or by concurrent chemoradiation in cases where resection was not feasible. This updated report delineates the 5-year overall survival (OS) and progression-free survival (PFS) rates.

**Result:**

Surgery was performed on 27 patients (57.4%), and R0 resection was observed in 26 patients (53.2%). Pathologic complete response was confirmed in four patients (8.5%). Following a minimum follow-up period exceeding 60 months for all patients, the total number of deaths was 31 (65.96%). The OS and PFS for the R0 group were significantly longer than those for the non-R0 group (median OS: 53.0 months vs. 13.9 months, HR: 0.36, 95% confidence interval [CI]: 0.17–0.76, *P* = 0.0032; median PFS: 50.84 months vs. 5.42 months, HR: 0.40, 95% CI 0.19–0.84, *P* = 0.0076). The 5-year OS rate was 50.0% (34.0%–73.4%) for the R0 group compared to 19.0% (7.9%–46.0%) for the non-R0 group (HR: 0.36, 95% CI: 0.17–0.79, *P* = 0.0041).

**Conclusion:**

Long-term follow-up confirmed that the OS and PFS were significantly improved in patients who underwent R0 resection compared to those who did not. The 5-year OS rate for patients who achieved R0 resection was 50.0%. R0 resection might be an independent prognostic factor for OS. To further improve the R0 resection rate and prognosis, more effective induction treatment regimens need to be explored.

**HIGHLIGHTS**

- For patients with BR-ESCC, OS and PFS were significantly improved in patients who underwent R0 resection compared to those who did not.
- The 5-year OS rate for patients who achieved R0 resection was 50.0%.
- R0 resection might be an independent prognostic factor for OS.
- R0 resection might be helpful in reducing the progression rate or delaying the progression in patients with BR-ESCC.

## Introduction

Esophageal cancer is a significant global health issue, ranking eleventh in incidence and seventh in mortality among all cancers^[^1^]^. Esophageal squamous cell carcinoma (ESCC) represents the predominant histological subtype in China^[^2,3^]^. Presently, perioperative multidisciplinary comprehensive treatment, including neoadjuvant chemoradiotherapy (NCRT), neoadjuvant chemotherapy (NCT), and adjuvant immunotherapy, is extensively implemented for locally advanced resectable ESCC, while definitive chemoradiotherapy (DCRT) is the standard treatment for unresectable ESCC^[^4–7^]^.

Due to the absence of a serosal layer in the esophagus, tumors originating from the esophageal mucosa can readily invade adjacent organs and structures, including the aorta, arch vessels, airways, vertebral body, and pericardium. Borderline resectable esophageal squamous cell carcinoma (BR-ESCC) is characterized by tumors or lymphadenopathy that are suspected of invading adjacent organs but cannot be definitively classified as T4b. Patients with BR-ESCC may still have the opportunity to undergo conversion surgery and achieve R0 resection following induction therapy. Several exploratory studies have reported on various treatment regimens for BR-ESCC patients, encompassing definitive chemoradiotherapy, induction chemoradiotherapy, and induction chemotherapy, demonstrating a 3-year overall survival rate of 40–70%^[^8–11^]^.

We conducted the NEOCRTEC1601 trial^[^12,13^]^ to assess the efficacy and safety of paclitaxel, cisplatin, and 5-fluorouracil (TPF) as induction chemotherapy for BR-ESCC. Between July 2014 and February 2019, a total of 47 patients diagnosed with BR-ESCC at Sun Yat-sen University Cancer Center were enrolled in the study. Preliminary results indicated that 53.2% of patients achieved R0 resection, and 8.5% of patients were confirmed to have a pathological complete response (pCR). The 3-year overall survival rate for all patients was 54.4%, and the 3-year overall survival rate for the patients who achieved R0 resection was 65.4%. The present article aims to report the 5-year overall survival rate and progression patterns observed in the study.

## Method

The study received approval from the Ethics Committee of Sun Yat-sen University Cancer Center. Prior to enrollment, all participants provided written informed consent. Additionally, the study was registered on ClinicalTrials.gov.

### STROCSS statement

The work has been reported in line with the STROCSS criteria^[^14^]^.

### Patients

The study enrolled patients with histologically confirmed BR-ESCC. BR-ESCC was characterized by primary tumors or bulky lymphadenopathy suspected of invading adjacent structures, including the aorta, arch vessels, airways, and vertebrae. Specific diagnostic methods included the following: Computed Tomography (CT) scans revealed that the adipose tissue between the tumor and the aorta appeared blurred, with the angle between the aorta and the tumor exceeding 90 degrees across three consecutive 2 mm slices. Alternatively, endoscopic ultrasound (EUS) indicated that the tumor had penetrated the outer layer of the esophagus, resulting in an indistinct boundary with the aorta. Additionally, endobronchial ultrasound (EBUS) demonstrated that the tumor was not clearly demarcated from the trachea or bronchus, although it did not invade the mucosa or submucosa of these structures. Other eligibility and exclusion criteria have been described in our previous articles^[^12,13^]^.

### Study design and end points

The NEOCRTEC1601 study was a single-center, prospective, phase II clinical trial. The primary endpoint was the R0 resection rate, while the secondary endpoints included overall survival (OS), progression-free survival (PFS), postoperative complications, pathological response, and adverse events. OS was defined as the duration from the registration date to the date of death or the last follow-up visit. PFS was defined as the duration from the registration date to the date of disease progression or death. This study aimed to report the 5-year OS rate, 5-year PFS rate, and progression patterns of the trial.

### Treatment procedure

Patients diagnosed with BR-ESCC received 2–3 cycles of induction chemotherapy every 3 weeks, consisting of paclitaxel (135 mg/m^2^ IV on day 1), cisplatin (75 mg/m^2^ IV on day 1), and TPF (4 g/m^2^ continuous infusion for 120 hours from day 1 to day 5). Subsequently, radical resection was performed 3–4 weeks after the completion of induction therapy, contingent upon the tumor being deemed resectable following a multidisciplinary consultation reassessment.

For patients with unresectable tumors after reassessment or those who underwent R1/R2 resection, concurrent chemoradiotherapy was recommended. A total dose of 56–60 Gy was administered in 2 Gy fractions per session. All patients received external beam radiation therapy using 6–8 MV photon beam with intensity-modulated radiation therapy. The gross tumor volume (GTV) encompassed the primary tumor and enlarged regional lymph nodes, as identified by contrast-enhanced CT scans, gastrointestinal barium studies, and esophageal endoscopy. The clinical target volume (CTV) was delineated by expanding the GTV with an additional lateral margin of 0.8–1.0 cm and a craniocaudal margin of 3.0–5.0 cm to account for potential subclinical disease involvement. The planning target volume (PTV) was delineated by expanding the CTV with a margin of 0.5–0.8 cm. The chemotherapy regimen depended on the tumor’s response to induction treatment. In cases where no progression was observed, a combination of paclitaxel and cisplatin was recommended.

### Surgery and pathologic analysis

An open or minimally invasive McKeown esophagectomy, combined with a two-field lymphadenectomy, was conducted. The severity of postoperative complications was classified according to the Clavien–Dindo classification system. Postoperative pathological assessments were independently carried out by two pathologists. pCR was defined as the absence of both grossly and microscopically viable tumor cells in the entire surgical specimen, including the primary site and any resected lymph nodes. Residual tumor status was classified according to the standard R-classification system: R0 indicated no residual tumor; R1 indicated microscopic residual tumor; and R2 indicated macroscopic residual tumor.

### Follow-up and progression

Patients were reviewed at three-month intervals during the first year, followed by six-month intervals for the subsequent 4 years, and annually thereafter until either death or the conclusion of the study. During each follow-up visit, evaluations comprised clinical examination, standard blood tests, cervical ultrasonography, and chest/abdominal CT and/or positron emission tomography (PET). Additionally, esophagogastroduodenoscopies (EGDs) with biopsies were conducted annually for a duration of 5 years.

The disease progression encompassed locoregional progression and distant progression (metastasis). Locoregional progression was characterized as relapse or progression occurring within the esophagus or regional lymph nodes, excluding the supraclavicular nodes. Distant metastasis was defined as the dissemination of disease to distant sites beyond the locoregional areas.

### Statistical analysis

The patients were stratified into two groups based on the status of R0 resection. The R0 group consisted of individuals who successfully underwent R0 resection. Conversely, the non-R0 group included patients who either did not undergo surgery (*n* = 20) or underwent surgery without achieving R0 resection (*n* = 1). OS and disease-free survival (DFS) were assessed using the Kaplan-Meier method, and intergroup comparisons were performed utilizing the log-rank test. The chi-square test or Fisher’s exact test was employed to evaluate differences in progression patterns. Additionally, the chi-square test and Student’s *t*-test were utilized to analyze the differences in clinical features between surgical and non-surgical patients. Two-sided *p*-values less than 0.05 were considered statistically significant. All statistical analyses were performed using R version 4.3.2 (The R Project for Statistical Computing, Vienna, Austria) and IBM SPSS version 29.0 (IBM, Chicago, IL, USA).

## Results

### Patients

From July 2014 to February 2019, a total of 47 eligible patients diagnosed with BR-ESCC at the Sun Yat-sen University Cancer Center were enrolled in the study. Table 1 presents the baseline characteristics of all patients. No statistically significant differences were observed between the surgical and non-surgical groups in terms of age, sex, tumor location, tumor differentiation, and clinical stage. Detailed information regarding treatment profiles, accrual procedures, safety profiles, surgery, and postoperative complication was presented in our previous report^[^13^]^. A total of 26 patients were confirmed to have undergone R0 resection, resulting in an R0 resection rate of 53.2%. Additionally, pCR was achieved in 4 patients, corresponding to a rate of 8.5%.

**Table 1:** Baseline characteristics of all patients

Characteristic	*N* (*n* = 47, %)	Surgery group (*n* = 27, %)	Non-surgery group (*n* = 20, %)	*P* value
Age (years)				0.819
Median	60	61	60	
Range	45–70	45–70	46–69	
Sex				0.534
Male	38 (80.9)	21 (77.8)	17 (85.0)	
female	9 (19.1)	6 (22.2)	11 (55.0)	
ECOG score				0.905
0	31 (66.0)	18 (66.7)	13 (65.0)	
1	16 (34.0)	9 (33.3)	7 (35.0)	
Tumor location				0.821
Upper	4 (8.5)	2 (7.4)	2 (10.0)	
Middle	29 (61.7)	17 (63.0)	12 (60.0)	
Lower	14 (29.8)	8 (29.6)	6 (30.0)	
Tumor differentiation				0.229
Well	2 (4.3)	2 (7.4)	0 (0)	
Moderate	32 (68.1)	16 (59.3)	16 (80.0)	
Poor	13 (27.7)	9 (33.3)	4 (20.0)	
Clinical T stage				0.202
3	4 (8.5)	2 (7.4)	2 (10.0)	
4a	28 (59.6)	19 (70.4)	9 (45.0)	
4b	15 (31.9)	6 (22.2)	9 (45.0)	
Clinical N stage				0.330
0	2 (4.3)	0	2 (10.0)	
1	22 (46.8)	13 (48.1)	9 (45.0)	
2	15 (31.9)	10 (37.0)	5 (25.0)	
3	8 (17.0)	4 (14.8)	4 (20.0)	
T stage after chemotherapy				<0.001
2	3 (6.4)	3 (11.1)	0	
3	25 (53.2)	22 (81.5)	3 (15.0)	
4a	12 (25.5)	1 (3.7)	11 (55.0)	
4b	7 (14.9)	1 (3.7)	6 (20.0)	
N stage after chemotherapy				0.210
0	3 (6.4)	2 (7.4)	1 (5.0)	
1	26 (55.3)	18 (66.7)	8 (40.0)	
2	14 (29.8)	6 (22.2)	8 (40.0)	
3	4 (8.5)	1 (3.7)	3 (15.0)	

### Survival

**Figure 1.**
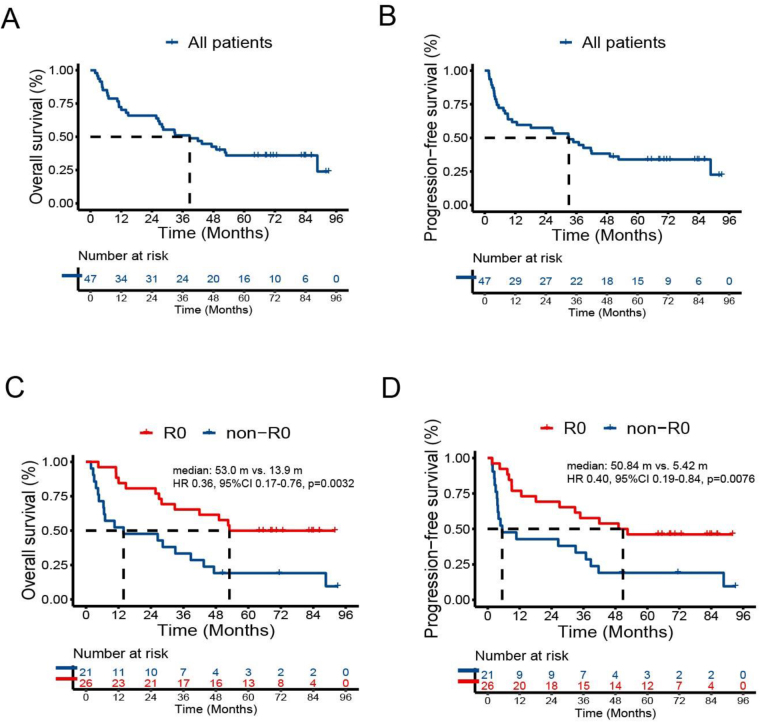
Overall survival and progression-free survival. A. OS of all patients. B. PFS of all patients. C. OS of R0 and non-R0 resection groups. D. PFS of R0 and non-R0 resection groups. OS, overall survival; PFS, progression-free survival.

Following a minimum follow-up period exceeding 60 months for all patients, the total number of deaths was 31 (65.96%). The median OS for all patients was 38.73 months (95% confidence interval [CI]: 18.37–59.08 months), and the median PFS for all patients was 33.04 months (95% CI: 19.04–47.04 months). The 5-year OS rate was 35.9% (24.5–52.7%), and the 5-year PFS rate was 33.9% (22.7–50.6%) (Fig. 1A,B).

The OS and PFS for the R0 group were significantly longer than the non-R0 group (median OS: 53.0 months vs. 13.9 months, HR: 0.36, 95% CI 0.17–0.76, *P* = 0.0032; median PFS: 50.84 months vs. 5.42 months, HR: 0.40, 95% CI 0.19–0.84, *P* = 0.0076) (Fig. 1C,D). The 5-year OS rate was 50.0% (34.0–73.4%) for the R0 group compared to 19.0% (7.9–46.0%) for the non-R0 group (HR: 0.36, 95% CI: 0.17–0.79, *P* = 0.0041). The 5-year PFS rate was 46.2% (30.5–69.9%) in the R0 group compared to 19.0% (7.9–46.0%) in the non-R0 group (HR: 0.41, 95% CI 0.19–0.86, *P* = 0.0098). Multivariable analysis revealed that R0 resection was the independent prognostic factors for OS (Table 2).

**Table 2:** Univariate and multivariate Cox regression analysis of prognostic factors in BR-ESCC

	Univariate analysis		Multivariate analysis	
Predictor	HR (95% CI)	*P* value	HR (95%CI)	*P* value
Age (years)				
<60				
≥60	1.03 (0.51–2.09)	0.93	1.01 (0.44–2.22)	0.97
Sex				
Male				
Female	0.39 (1.12–1.29)	0.12	0.55 (0.13–2.39)	0.43
Smoking history				
No				
Yes	0.74 (0.82–3.70)	0.15	1.50 (0.53–4.22)	0.44
cN stage				
0–1				
2–3	1.26 (0.61–2.59)	0.52	1.10 (0.52–2.33)	0.80
Cycles of induction therapy				
1–2				
3–4	1.18 (0.57–2.45)	0.66	1.80 (0.78–4.15)	0.17
Stage after induction				
I–III				
IV	2.03 (0.99–4.13)	0.05	1.37 (0.25–2.17)	0.57
pCR or not				
No				
Yes	0.25 (0.03–1.83)	0.17	0.31 (0.04–2.50)	0.27
R0 resection				
No				
Yes	0.35 (0.17–0.72)	0.003	0.29 (0.09–0.92)	0.036

### Progression patterns

Following a median follow-up period of 50.49 months (range: 6.94–94.51 months) for the entire cohort and 82.94 months for survivors, disease progression was observed in 14 patients (53.8%) in the R0 resection group compared to 18 patients (85.7%) in the non-R0 group (*P* = 0.02). Within the R0 group, 5 patients (19.2%) exhibited distant metastasis, 2 patients (7.7%) demonstrated locoregional recurrence, 1 patient (3.8%) experienced concurrent distant and locoregional progression, and the progression patterns in 6 patients (23.1%) could not be definitively confirmed until the time of death. In the non-R0 group, distant metastasis was confirmed in 5 patients (23.8%), locoregional progression was confirmed in 3 patients (14.3%), and the progression patterns in 10 patients (47.6%) could not be determined until the time of death. No statistically significant differences were observed in the rates of confirmed distant metastasis (p = 0.56) and local recurrence (p = 0.61) between the two groups. The progression timing and frequency in accordance with treatment groups are detailed in Table 3, and the specific sites and organs involved in disease progression are shown in Table 4.

**Table 3:** progression timing and frequency in patients with BR-ESCC

Groups	Total	Progression	≤12.0 months	12.1–24.0 months	24.1–36.0 months	36.1–48.0 months	48.1–60 months	≥60.0 months
R0	26	14, 53.8%	6, 23.1%	2, 7.7%	2, 7.7%	2, 7.7%	2, 7.7%	0
Non-R0	21	18, 85.7%	12, 57.1%	0	2, 9.5%	3, 14.3%	0	1, 4.8%

**Table 4:** Disease progression sites and organs

Progression patterns	R0 group (*N* = 26, %)	Non-R0 group (*N* = 21, %)
Metastasis		
Non-regional lymph node	4 (15.4)	2 (9.5)
Lung	0	2 (9.5)
Liver	1 (3.8)	1 (4.8)
Vertebra	1 (3.8)	0
Locoregional progression		
Regional lymph nodes	1 (3.8)	0
Primary site	1 (3.8)	3 (14.3)
Anastomosis	1 (3.8)	0
Unknown	6(23.1)	10 (47.6)

## Discussion

At present, different treatment modalities are recommended for ESCC depending on the clinical staging of the disease. Conversion surgery after induction therapy represents a promising therapeutic approach for patients with borderline resectable esophageal cancer that has not been classified as cT4b. In our study, 47 BR-ESCC patients received induction chemotherapy with the TPF regimen. Ultimately, 26 (53.2%) patients achieved R0 resection, which met the primary endpoint of the study. Long-term follow-up revealed that the OS and PFS were significantly improved in patients who underwent R0 resection compared to those who did not. The 5-year OS rate for patients who achieved R0 resection was 50.0%. Multivariable analysis revealed that R0 resection might be an independent prognostic factor for OS. Moreover, our data revealed that R0 resection might be helpful in reducing the progression rate or delaying the progression in patients with BR-ESCC.

The DCF regimen has been extensively utilized in neoadjuvant and induction therapies within Japanese studies. In the JCOG-1109 trial, the DCF group achieved a 5-year OS rate of 65.1% among patients with stage IB/II/III ESCC, which was significantly superior to the doublet regimen^[^15^]^. However, the JCOG-1109 trial did not enroll patients with BR-ESCC. The COSMOS study, which recruited 48 patients with unresectable or borderline-resectable ESCC, assessed the safety and efficacy of the DCF regimen as induction chemotherapy. In the study, R0 resection was achieved in 19 patients (39.6%), and definitive CRT after induction chemotherapy did not significantly improve the conversion surgery rate of the patients (only increasing by 1 patient). The follow-up results indicated that the prognosis for patients who underwent R0 resection was better than for those who did not, with a reported 3-year OS rate of 71.4% for patients with R0 resection. However, the 5-year follow-up results were not reported. Another retrospective study conducted by Nakajima, *et al*^[^16^]^ analyzed 32 patients with borderline-resectable cT3 tumors and administered 2 cycles of DCF induction therapy. The findings revealed an R0 resection rate of 81.5%. The 5-year OS rate for all patients was 46.4%, and 55.0% for those who underwent conversion surgery. However, the retrospective study had a large tendency for selection bias, and the small sample size necessitates cautious interpretation of the results. In summary, induction chemotherapy has demonstrated efficacy and safety as a treatment strategy for patients with BR-ESCC. To our knowledge, no prospective study has yet reported the 5-year OS rate for patients with BR-ESCC. In our prospective trial, the 5-year OS rate for patients who underwent R0 resection was 50%, a figure that aligns with findings from other retrospective studies. The 5-year survival rate declined to 50%, attributed to disease progression and subsequent mortality in 4 patients during the follow-up period between the 3rd and 5th years.

Preoperative chemoradiotherapy has been shown to enhance local tumor control rates and increase the likelihood of achieving radical resection, which has become the standard treatment option for locally advanced resectable ESCC^[^17,18^]^, and achieved a 5-year OS rate of 59.9%^[^19^]^. Preoperative chemoradiotherapy has been considered an alternative treatment option for patients with BR-ESCC. Several previous retrospective studies have reported 5-year OS rates. A retrospective study performed by Toshihiko, *et al*^[^20^]^ aimed to compare the clinical benefits of NCT versus NCRT in patients with esophageal cancer (including partial T4 patients). The survival results demonstrated that the 5-year OS rates for borderline-resectable T4 patients who underwent NCT and NCRT were 0% and 27.8%, respectively. However, the number of borderline-resectable patients in this study was relatively small (n = 17), which was not compelling. Another retrospective study^[^21^]^ compared salvage surgery after definitive chemoradiotherapy (SALV) versus neoadjuvant chemoradiotherapy plus surgery (NCRS) in patients with BR-ESCC. The findings indicated similar R0 resection rate (SALV vs NCRS: 81% vs. 85%, *P* = 0.25) and 5-year OS rate (SALV vs. NCRS: 26% vs 27%) of two groups. In a study conducted by Yoshihiro, *et al*^[^22^]^ =the treatment efficacy of preoperative chemoradiotherapy in patients with borderline-resectable and resectable cT3 (cT3br, cT3r) esophageal cancer was evaluated. Among the 68 cT3br patients, 94.8% achieved R0 resection and 12.1% achieved pCR, with a reported 5-year OS rate of 56.8%. Additionally, in another study reported by Ryosuke, *et al*^[^23^]^, the 5-year OS rate of borderline-resectable cT3 patients treated with NCRT followed by surgery was 38.4%. However, the majority of studies were retrospective analyses, with a large tendency for selection bias. In addition, for patients who were defined as unresectable after induction chemoradiotherapy, subsequent treatment options remained limited. For those who underwent R1 or R2 resection, the dose and field of induction radiation were found to be inadequate, thereby elevating the risk of disease progression. Conversely, in our treatment approach, patients who were not successful in undergoing conversion surgery following induction chemotherapy still have the opportunity to receive definitive CRT. Furthermore, induction chemotherapy serves to assess the sensitivity of tumors to the TPF chemotherapy regimen.

DCRT was the standard treatment for patients with unresectable ESCC. A retrospective study by Ishikura, *et al*^[^10^]^ evaluated the therapeutic effects of DCRT in BR-ESCC patients, reporting a 3-year OS rate of 46%. However, no studies have reported the 5-year OS rate for DCRT in BR-ESCC patients. JCOG-1510^[^24^]^, a randomized phase III trial, aim to confirm the superiority of induction chemotherapy with docetaxel plus cisplatin and TPF followed by conversion surgery or definitive CRT over definitive CRT alone for OS in patients with locally advanced unresectable squamous-cell carcinoma of the thoracic esophagus. Besides, DCRT in combination with conversion surgery may represent a viable alternative treatment option. We recommend that this hypothesis be rigorously investigated in future research.

Enhancing the conversion rate and the R0 resection rate represents a significant focus for future research endeavors in BR-ESCC. Immunotherapy, particularly immune checkpoint inhibitors, has emerged as a prominent and effective treatment modality for advanced esophageal cancer. Furthermore, several studies have demonstrated the efficacy of immunotherapy in perioperative treatment^[^25–27^]^. Chao *et al*^[^28^]^ retrospectively analyzed 27 patients with borderline-resectable or unresectable ESCC who received preoperative camrelizumab plus albumin-bound paclitaxel and carboplatin. The results indicated that 19 patients (70.4%) achieved R0 resection, and 7 patients (25.9%) attained pCR. Based on the treatment efficacy of sintilimab in patients with advanced ESCC^[^29^]^, our center is conducting a phase II clinical trial to investigate whether induction immunochemotherapy (sintilimab combined with albumin-bound paclitaxel and cisplatin) can enhance the rate of R0 resection and pCR in patients with BR-ESCC.

### Limitation

The study has several limitations. First, the progression data were insufficient to adequately demonstrate the details of progression patterns. Second, the sample size was small, and the treatment regimens were not consistently implemented as planned due to issues with patient compliance and other factors. Third, our study did not include patients with esophageal adenocarcinoma. Consequently, the conclusions drawn from this study should be interpreted with caution.

## Conclusion

Long-term follow-up evaluation confirmed that the OS and PFS were significantly improved in patients who underwent R0 resection compared to those who did not. The 5-year OS rate for patients who achieved R0 resection was 50.0%. R0 resection might be an independent prognostic factor for OS. To further improve the R0 resection rate and prognosis, more effective induction treatment regimens need to be explored.

## Data Availability

Publicly available.

## Research registration unique identifying number (UIN)

The study was registered at ClinicalTrials.gov (NCT02976909).

## References

[1] Bray F Laversanne M Sung H. Global cancer statistics 2022: GLOBOCAN estimates of incidence and mortality worldwide for 36 cancers in 185 countries. CA Cancer J Clin 2024;74:229–63. 38572751 10.3322/caac.21834

[2] Abnet CC Arnold M Wei WQ. Epidemiology of esophageal squamous cell carcinoma. Gastroenterology 2018;154:360–73. 28823862 10.1053/j.gastro.2017.08.023 PMC5836473

[3] Zheng RS Chen R Han BF. Cancer incidence and mortality in China, 2022. Zhonghua Zhong Liu Za Zhi 2024;46:221–31. 38468501 10.3760/cma.j.cn112152-20240119-00035

[4] Center CN. Chinese guidelines on perioperative management of resectable esophageal cancer (2023 edition). Zhonghua Yi Xue Za Zhi 2023;103:2552–70. 37650202 10.3760/cma.j.cn112137-20230604-00933

[5] Shah MA Kennedy EB Catenacci DV. Treatment of locally advanced esophageal carcinoma: ASCO guideline. J Clin Oncol 2020;38:2677–94. 32568633 10.1200/JCO.20.00866

[6] Ajani JA D’Amico TA Bentrem DJ. Esophageal and esophagogastric junction cancers, version 2.2023, NCCN clinical practice guidelines in oncology. J Natl Compr Canc Netw 2023;21:393–422. 37015332 10.6004/jnccn.2023.0019

[7] Obermannová R Alsina M Cervantes A. Oesophageal cancer: ESMO clinical practice guideline for diagnosis, treatment and follow-up. Ann Oncol 2022;33:992–1004. 35914638 10.1016/j.annonc.2022.07.003

[8] Yokota T Kato K Hamamoto Y. A 3-year overall survival update from a phase 2 study of chemoselection with DCF and subsequent conversion surgery for locally advanced unresectable esophageal cancer. Ann Surg Oncol 2020;27:460–67. 31376034 10.1245/s10434-019-07654-8

[9] Suzuki T Okamura A Watanabe M. Neoadjuvant chemoradiotherapy with cisplatin plus fluorouracil for borderline resectable esophageal squamous cell carcinoma. Ann Surg Oncol 2020;27:1510–17. 31820213 10.1245/s10434-019-08124-x

[10] Ishikura S Kondo T Murai T. Definitive chemoradiotherapy for squamous cell carcinoma of the esophagus: outcomes for borderline-resectable disease. J Radiat Res 2020;61:464–69. 32249307 10.1093/jrr/rraa008 PMC7299256

[11] Yokota T Kato K Hamamoto Y. Phase II study of chemoselection with docetaxel plus cisplatin and 5-fluorouracil induction chemotherapy and subsequent conversion surgery for locally advanced unresectable oesophageal cancer. Br J Cancer 2016;115:1328–34. 27811857 10.1038/bjc.2016.350 PMC5129815

[12] Wang Z Hu M Hu Y. Paclitaxel plus cisplatin and 5-fluorouracil induction chemotherapy for locally advanced borderline-resectable esophageal squamous cell carcinoma: a phase II clinical trial. Esophagus 2022;19:120–28. 34319435 10.1007/s10388-021-00864-8

[13] Wu JD Wang ZQ Li QQ. A 3-year survival update from a phase 2 study of paclitaxel plus cisplatin and 5-fuorouracil induction chemotherapy for locally advanced borderline-resectable esophageal squamous cell carcinoma: the NEOCRTEC-1601 clinical trial. Ann Surg Oncol 2024;31:838–46. 37919448 10.1245/s10434-023-14513-0 PMC10761379

[14] Rashid R Sohrabi S Kerwan A Franchi T Mathew G Nicola M. Agha R for the STROCSS Group. The STROCSS 2024 guideline: strengthening the reporting of cohort, cross-sectional, and case-control studies in surgery. Int J Surg 2024;110:3151–65. 38445501 10.1097/JS9.0000000000001268 PMC11175759

[15] Kato K Ito Y Daiko H. A phase III trial comparing 5-FU plus cisplatin (CF) versus CF plus docetaxel or radiotherapy as neoadjuvant treatment for locally advanced esophageal cancer: 5-year follow-up from JCOG1109. J Clin Oncol 2024;42:4082–.

[16] Nakajima M Muroi H Kikuchi M. Therapeutic strategy aiming at R0 resection for borderline-resectable esophageal squamous cell carcinoma using induction chemotherapy with docetaxel, cisplatin, and 5-fluorouracil. Gen Thorac Cardiovasc Surg 2023;71:584–90. 37060435 10.1007/s11748-023-01934-7

[17] Yang H Liu H Chen Y. Neoadjuvant chemoradiotherapy followed by surgery versus surgery alone for locally advanced squamous cell carcinoma of the esophagus (NEOCRTEC5010): a phase III multicenter, randomized, open-label clinical trial. J Clin Oncol 2018;36:2796–803. 30089078 10.1200/JCO.2018.79.1483 PMC6145832

[18] van Hagen P Hulshof MC van Lanschot JJ. Preoperative chemoradiotherapy for esophageal or junctional cancer. N Engl J Med 2012;366:2074–84. 22646630 10.1056/NEJMoa1112088

[19] Yang H Liu H Chen Y. Long-term efficacy of neoadjuvant chemoradiotherapy plus surgery for the treatment of locally advanced esophageal squamous cell carcinoma: the NEOCRTEC5010 randomized clinical trial. JAMA Surg 2021;156:721–29. 34160577 10.1001/jamasurg.2021.2373 PMC8223138

[20] Nakashima Y Saeki H Hu Q. Neoadjuvant chemotherapy versus chemoradiotherapy for patients with esophageal squamous cell carcinoma. Anticancer Res 2018;38:6809–14. 30504394 10.21873/anticanres.13053

[21] Shiraishi O Yasuda T Kato H. Comparison of aggressive planned salvage surgery versus neoadjuvant chemoradiotherapy plus surgery for borderline resectable T4 squamous cell carcinoma. Ann Surg Oncol 2021;28:6366–75. 33768398 10.1245/s10434-021-09875-2

[22] Wakita A Motoyama S Sato Y. Preoperative neoadjuvant chemoradiotherapy provides borderline resectable thoracic esophageal cancer with equivalent treatment results as clinically T3 thoracic esophageal cancer. Ann Gastroenterol Surg 2023;7:904–12. 37927919 10.1002/ags3.12706 PMC10623951

[23] Hirohata R Hamai Y Hihara J. Evaluation of neoadjuvant chemoradiotherapy followed by surgery for borderline resectable esophageal squamous cell carcinoma. World J Surg 2022;46:1934–43. 35508816 10.1007/s00268-022-06568-z

[24] Terada M Hara H Daiko H. Phase III study of tri-modality combination therapy with induction docetaxel plus cisplatin and 5-fluorouracil versus definitive chemoradiotherapy for locally advanced unresectable squamous-cell carcinoma of the thoracic esophagus (JCOG1510: tRIANgLE). Jpn J Clin Oncol 2019;49:1055–60. 31411696 10.1093/jjco/hyz112

[25] Chen R Liu Q Li Q. A phase II clinical trial of toripalimab combined with neoadjuvant chemoradiotherapy in locally advanced esophageal squamous cell carcinoma (NEOCRTEC1901). EClinicalMedicine 2023;62:102118. 37560259 10.1016/j.eclinm.2023.102118 PMC10407031

[26] Kelly RJ Ajani JA Kuzdzal J. Adjuvant nivolumab in resected esophageal or gastroesophageal junction cancer. N Engl J Med 2021;384:1191–203. 33789008 10.1056/NEJMoa2032125

[27] Qin J Xue L Hao A Neoadjuvant chemotherapy with or without camrelizumab in resectable esophageal squamous cell carcinoma: the randomized phase 3 ESCORT-NEO/NCCES01 trial. Nature Med 2024;30:2549-57. 38956195 10.1038/s41591-024-03064-w PMC11405280

[28] Chao L Liu J Chen Y Fan Y Guo S Zhang S. Benefits of camrelizumab plus carboplatin and albumin paclitaxel as induction therapy for locally advanced borderline resectable or unresectable esophageal squamous cell carcinoma. Thorac Cancer 2024;15:622–29. 38316630 10.1111/1759-7714.15232 PMC10928255

[29] Lu Z Wang J Shu Y. Sintilimab versus placebo in combination with chemotherapy as first line treatment for locally advanced or metastatic oesophageal squamous cell carcinoma (ORIENT-15): multicentre, randomised, double blind, phase 3 trial. Bmj 2022;377:e068714. 35440464 10.1136/bmj-2021-068714 PMC9016493

